# Frequent gain- and loss-of-function mutations of the *BjMYB113* gene accounted for leaf color variation in *Brassica juncea*

**DOI:** 10.1186/s12870-021-03084-5

**Published:** 2021-06-29

**Authors:** Guanghui An, Jiongjiong Chen

**Affiliations:** Key Laboratory of Horticultural Plant Biology, Ministry of Education, Key Laboratory of Horticultural Crop Biology and Genetic Improvement (Central Region), MOA, College of Horticulture and Forestry Sciences, Huazhong Agricultural University, 430070 Wuhan, People’s Republic of China

**Keywords:** Leaf color, MYB transcription factor, BSR-seq, Map-based cloning, *Brassica juncea*

## Abstract

**Background:**

Mustard (*Brassica juncea*) is an important economic vegetable, and some cultivars have purple leaves and accumulate more anthocyanins than the green. The genetic and evolution of purple trait in mustard has not been well studied.

**Result:**

In this study, free-hand sections and metabolomics showed that the purple leaves of mustard accumulated more anthocyanins than green ones. The gene controlling purple leaves in mustard, *Mustard Purple Leaves* (*MPL*), was genetically mapped and a *MYB113-like* homolog was identified as the candidate gene. We identified three alleles of the *MYB113-like* gene, *BjMYB113a* from a purple cultivar, *BjMYB113b* and *BjMYB113c* from green cultivars. A total of 45 single nucleotide polymorphisms (SNPs) and 8 InDels were found between the promoter sequences of the purple allele *BjMYB113a* and the green allele *BjMYB113b*. On the other hand, the only sequence variation between the purple allele *BjMYB113a *and the green allele *BjMYB113c* is an insertion of 1,033-bp fragment in the 3’region of *BjMYB113c*. Transgenic assay and promoter activity studies showed that the polymorphism in the promoter region was responsible for the up-regulation of the purple allele *BjMYB113a *and high accumulation of anthocyanin in the purple cultivar. The up-regulation of *BjMYB113a* increased the expression of genes in the anthocyanin biosynthesis pathway including *BjCHS*, *BjF3H*, *BjF3’H*, *BjDFR*, *BjANS* and *BjUGFT*, and consequently led to high accumulation of anthocyanin. However, the up-regulation of *BjMYB113* was compromised by the insertion of 1,033-bp in 3’region of the allele *BjMYB113c*.

**Conclusions:**

Our results contribute to a better understanding of the genetics and evolution of the *BjMYB113* gene controlling purple leaves and provide useful information for further breeding programs of mustard.

**Supplementary Information:**

The online version contains supplementary material available at 10.1186/s12870-021-03084-5.

## Background

Mustard (*Brassica juncea*, 2n = 36, AABB) is an important economic vegetable in the world [1, 2]. This cultivated species contains several different varieties, which exhibit extreme morphologic polymorphisms, with leaves, stems, roots, or seeds as harvest organs. For leafy mustard cultivars, some have green leaves while others have purple leaves. The purple color is caused by the accumulation of anthocyanins in leaf epidermal cells [3].

Anthocyanins are a large group of water-soluble natural pigments and widely distribute in higher plants. They are largely responsible for the red, purple, and blue colors in flowers, fruits, leaves, seeds and other plant organs [4, 5]. In plants, anthocyanins play important roles in attracting pollinators and seed distributors [5]. Anthocyanins can protect plants from UV damage, and they have strong capacity to scavenge reactive oxygen species (ROS), which is critical for biotic and abiotic tolerance [6–11]. This group of metabolites also promotes human health with obvious functions against obesity, inflammation, coronary arteriosclerosis, and cancers [12–16].

Many previous studies have shown that transcriptional regulation of anthocyanin biosynthesis in higher plants is mainly controlled by the MBW ternary complex containing MYB, bHLH and WD repeat transcription factors [17, 18]. For example, AtMYB113 forms a complex with a bHLH and a WD40 protein, which actives the genes in the anthocyanin biosynthetic pathway leading to pigment accumulation in *Arabidopsis thaliana* [19]. The MBW ternary complex transcriptionally regulates the genes in the anthocyanin biosynthetic pathway, including *phenylalanine-ammonia lyase* (*PAL*), *chalcone synthase* (*CHS*), *chalcone isomerase* (*CHI*), *flavanone 3-hydroxylase* (*F3H*), *dihydroflavonol 4-reductase* (*DFR*), and *anthocyanidin synthase* (*ANS*) [20, 21]. Upregulation of components of the MBW complex has been widely researched and shown to be responsible for high anthocyanin accumulation in many plant species such as peach [22], apple [23], pear [24], grape [25], blood orange [26], petunia [27], rice [28], lettuce [29], bokchoy [30], kale [31], cauliflower [32], barrel medic [33], etc. In addition to the MBW complex, other transcription factors, such as NACs, HY5, ERFs, BBX22, and WRKY, were found to be involved in anthocyanin biosynthesis as well. These transcription factors can bind to the promoters of component genes of the MBW complex indirectly or directly to regulate their expressions and consequently anthocyanin biosynthesis [22, 34, 35].

The genus Brassica harbors several important vegetable crops, such as cabbage, cauliflower, Chinese cabbage, and mustard, all with both green and purple cultivars. Purple is an attracting color in vegetables, and its underlying metabolites provide health benefits to consumers. In *Brassica rapa*, mutants of the *anl* locus and the *Anp* locus failed to produce anthocyanin pigments; *BrEGL3.1* and *BrEGL3.2*, two genes encoding bHLH transcription factors, promote anthocyanin biosynthesis in Chinese cabbage. The activated *BoMYB2* gene accounts for the anthocyanin accumulation in purple cabbage and cauliflower [31, 32]. Recently, a genetic analysis of the purple trait in mustard using an F_2_ population derived from a cross between a purple and a green cultivar suggested that an insertion in the first intron of a MYB-encoding gene (*BjPur*) changed the purple leaves to green [3]. However, the function of the *BjPur* gene in mustard has not been verified, and the genetic and molecular mechanisms underlying purple mustard remain unclear.

Compared with wild progenitors, cultivated horticultural crops showed rich color polymorphisms. The gain-of-function mutations on the anthocyanin pathway have mainly occurred in the encoding genes of members of the MBW complex (see above). For example, the *BoMYB2* gene in *Brassica oleracea* was activated independently for at least three times causing purple color in kale, cauliflower, and cabbage. The activation events in *BoMYB2* included an insertion of a CACTA transposon, an insertion of a harbinger transposon and point mutations in its promoter region, respectively [31, 32]. Four genes encoding transcription factors bHLH, R2R3-MYB, R3-MYB and WD40, respectively, showed sequence and functional polymorphisms in cultivated lettuce, and mutations on three of them promoted accumulation of anthocyanins on lettuce leaves [29].

In this study, we dissected the genetics controlling purple mustard using Bulked Segregant Analysis and RNA-seq (BSR-seq). The gene was genetically fine-mapped and functionally confirmed through agrobacteria-mediated transformation. The mutation events were analyzed in detail, and the evolution of the causal gene was proposed. Our study contributes to a better understanding of the genetics and evolution of the gene controlling purple leaves and provides useful information for further breeding programs of mustard.

## Results

### Characterization of anthocyanin in mustard cultivars

**Fig. 1:**
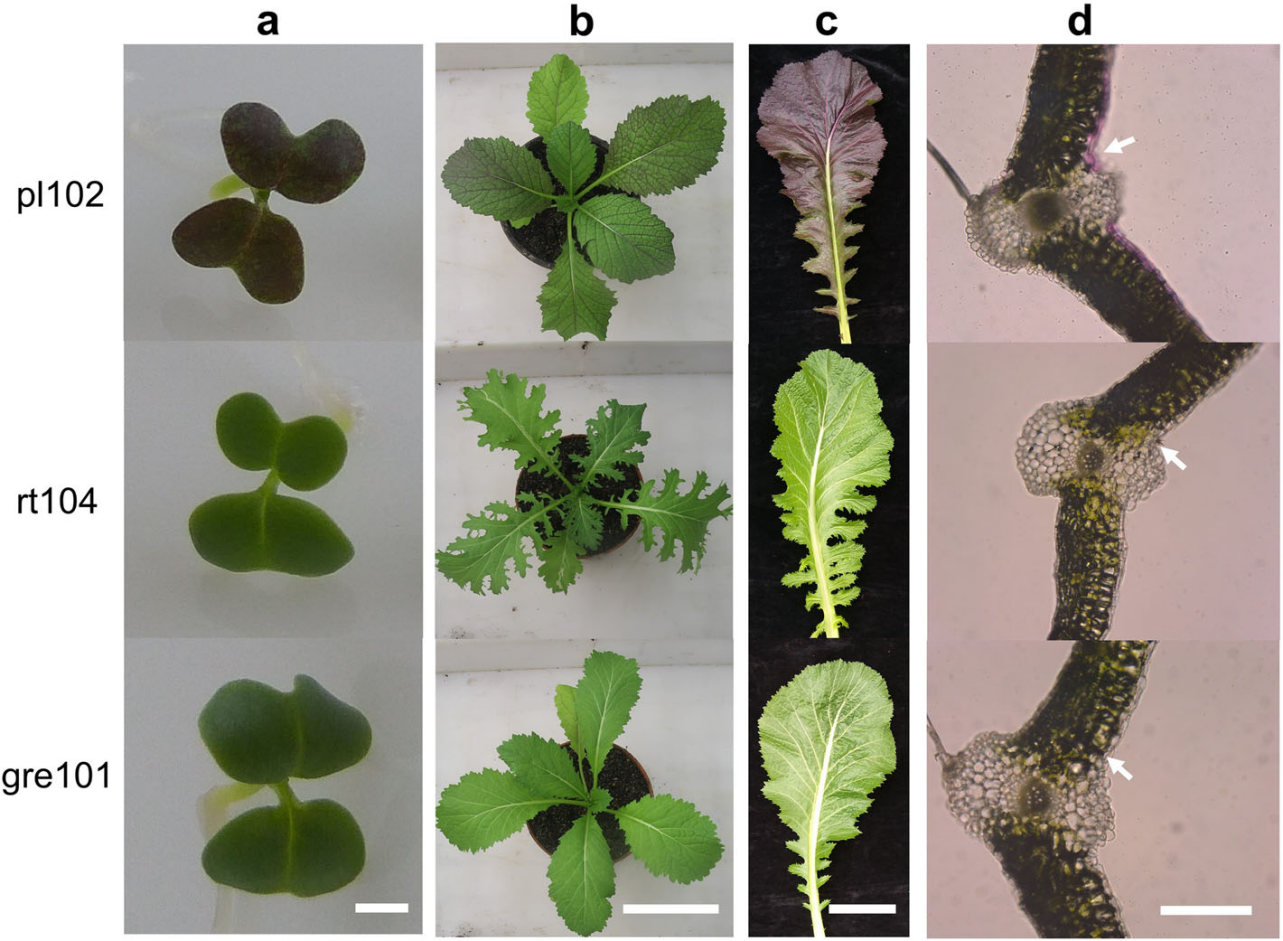
Color variation in three *Brassica juncea* cultivars, pl102, rt104, gre101. **a** Four-day-old seedlings for pl102, rt104 and gre101. Scale bar represents 1 mm. **b** One-month-old seedlings for pl102, rt104 and gre101. Scale bar represents 10 cm. **c** Three-month-old leaves for pl102, rt104 and gre101. Scale bar represents 10 cm. **d** Free-hand sections of leaf vein tissues for pl102, rt104 and gre101. The white arrows indicate that anthocyanins accumulated in the epidermal cells of purple leaves of pl102, but were absent in rt104 and gre101. Scale bar represents 0.1 mm

**Fig. 2:**
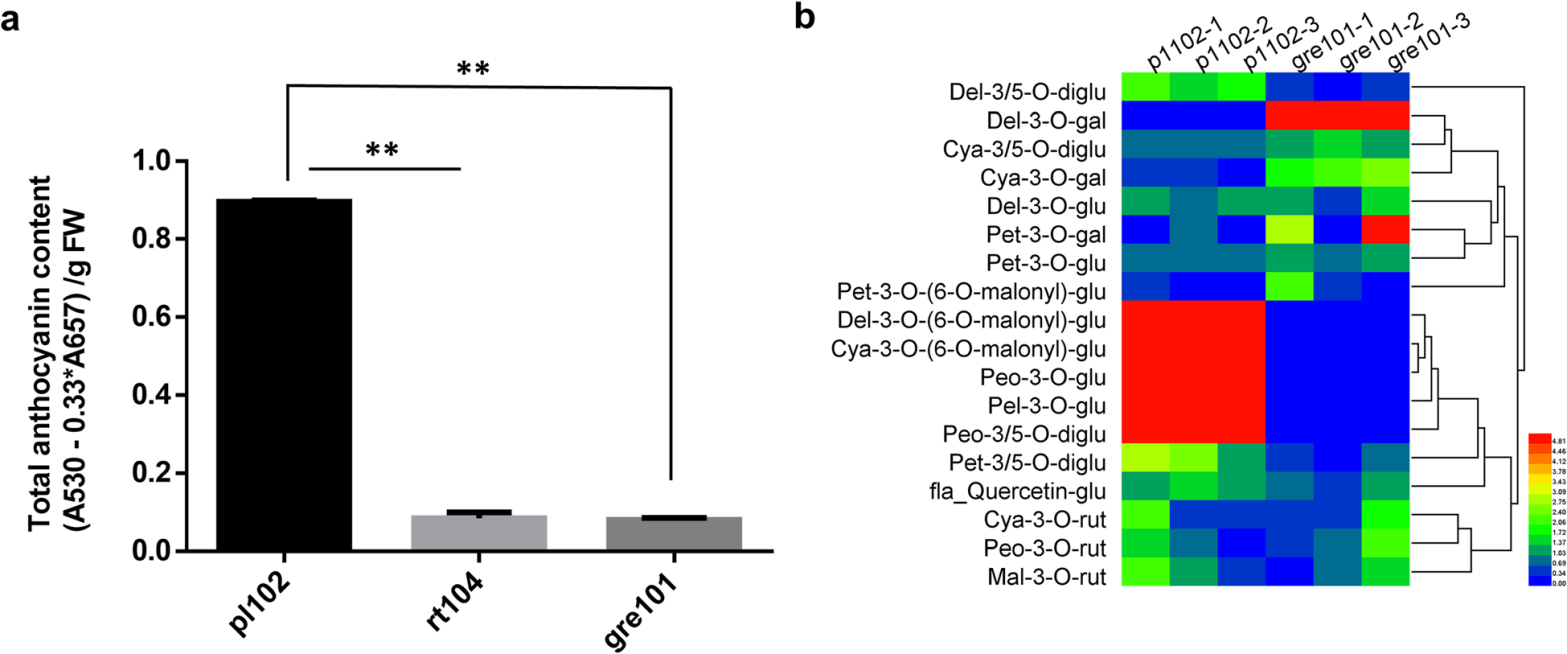
Analysis of total anthocyanin contents and anthocyanin compounds in purple and green parental mustard cultivars. **a** Total anthocyanin contents of purple and green cultivars (leaves). **b** Heatmap of anthocyanin compounds in purple (pl102) and green (gre101) cultivars with three biological replicates, respectively. The abbreviations of anthocyanin compounds were shown in the left, and the corresponding full name of anthocyanin compounds were list in the Table S1. Statistical analyzes were conducted by Student’s t test, **P < 0.01

Mustard has both green and purple cultivars (Fig. 1a, b, c). We compared the total anthocyanin contents of one purple mustard cultivar (pl102) and two green cultivars (rt104 and gre101). As expected, the total anthocyanin content in the purple cultivar was considerably higher than those in the two green cultivars (Fig. 2a). Free-hand sections of leaf tissues of the purple cultivar showed that the anthocyanins accumulated mainly in the epidermal cells and in less extent in the mesophyll cells near the epidermises. In contrast, no obvious anthocyanins were found in green cultivars (Fig. 1d).

We further used Liquid Chromatography-Electrospray Ionization-Mass Spectrometry (LC-ESI-MS) to analyze the types of anthocyanins and their concentrations in the purple and green cultivars. A total of 18 anthocyanins were identified (Table S1). Of them, six had significantly higher concentration in the purple cultivar than in green one (*p* < 0.05), including Delphinidin 3,5-O-diglucoside, Delphinidin 3-O-(6’’-O-malonyl)-beta-D-glucoside, Cyanidin 3-O-(6’’-O-malonyl)-beta-D-glucoside, Pelargonidin 3-O-glucoside, Peonidin 3,5-O-diglucoside, and Peonidin 3-O-glucoside, all emitted dark color (Fig. 2b). We conclude that the purple color in mustard leaves was caused by high accumulation of these anthocyanins.

### Map-based cloning of the gene controlling color variation in mustard

As mentioned in Background section, a previous study suggested that an insertion of 1,268-bp fragment in the first intron of a *MYB* gene was responsible for its loss-of-function and green leaves [3]. Furthermore, some green cultivars such as rt104 and gre101 do not have the 1,268-bp insertion, and therefore should have distinct mechanisms for loss-of-function. To answer these questions, purple cultivar pl102 was crossed with green cultivars rt104 and gre101, respectively. Both F_1_ hybrids were purple, and they were selfed to generate two F_2_ segregating populations.

**Fig. 3:**
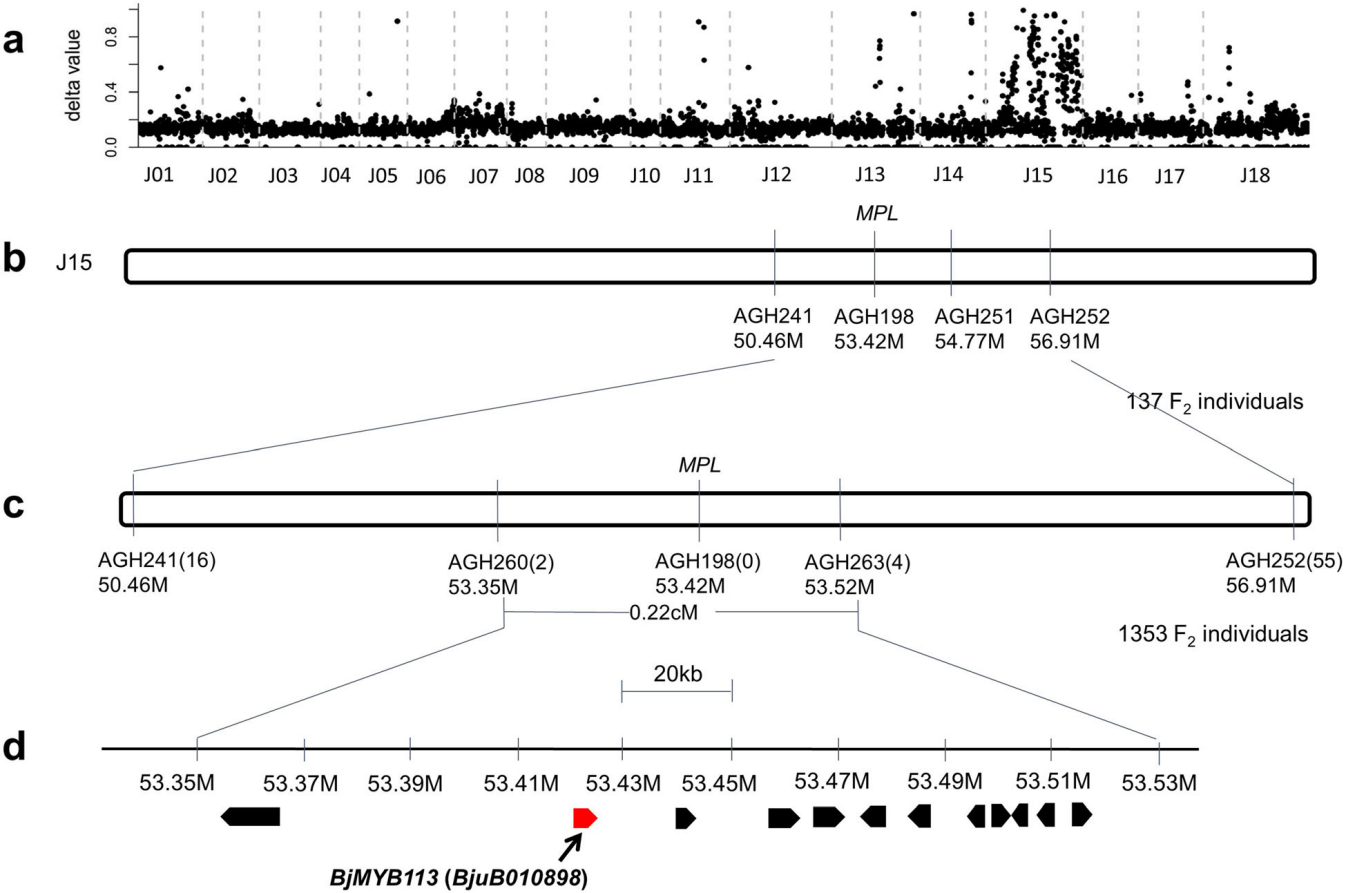
Map-based cloning of the *MPL* gene in the F_2_ population of pl102 × rt104. **a** The value of Δ(SNP-index) plotted along the eighteen chromosomes (X-axis) of mustard. High values were shown on Chromosome J15. **b** The *MPL* gene was mapped between markers AGH241 and AGH252 in the interval of 50.46 Mb-56.91 Mb on J15 using 137 individuals. **c** The *MPL* gene was fine mapped between markers AGH260 and AGH263 with the interval of 170 kb on J15 using 1,353 individuals. The numbers in parentheses refer to the number of recombinants. **d** Schematic diagram of predicted genes at the *MPL* locus. The broad arrows represent twelve predicted genes in the 170 kb region. The *BjMYB113* (*BjuB010898*) gene was in red broad arrow, considered as the candidate gene

**Fig. 4:**
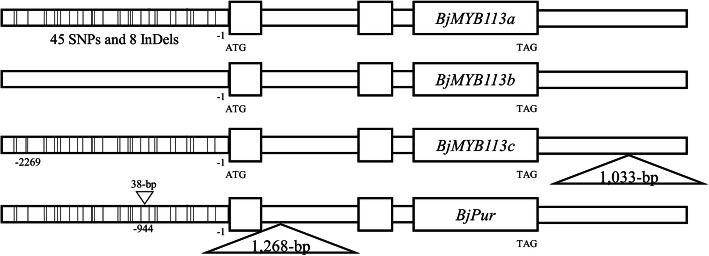
The variations of four *BjMYB113* alleles. The vertical lines in promotor region represent point mutations and InDels. The triangle in 3’region or intron represents fragment insertion

Of the 137 individuals in the F_2_ segregating population derived from the cross pl102 × rt104, 102 individuals had purple leaves and 35 individuals had green leaves, with an expected ratio of 3:1 (χ^2^ = 0.0219 < χ^2^ (0.05,1) = 3.84, *P* > 0.05, Table S2), suggesting a single gene controlling the color variation, which was referred to as *Mustard Purple Leaves* (*MPL*) in this study. Then, we used BSR-seq to genetically map the gene underlying the color variation. The *MPL* gene was mapped to chromosome J15 (B05) (Fig. 3a). Screening a total of 1,353 individuals from the F_2_ population ultimately mapped the *MPL* gene between markers AGH260 and AGH263 on J15 (B05), in an interval of approximately 170 kb region (Fig. 3b, c). Only 12 open reading frames (ORFs) were predicted in this interval. The *MYB* gene, which is an ortholog of *MYB113* in *Arabidopsis* and was previously shown to control color variation in mustard, was also located in the candidate interval and remained a reliable candidate gene (*BjMYB113*) (Fig. 3d). As mentioned above, the 1,268-bp insertion, which was suggested to inactivate the candidate gene in a previous study, was absent in the green parent [3]. To verify the *BjMYB113* as the candidate gene, we first investigated its sequence variation between the two parents, including its 2,842 bp upstream sequences. A total of 45 SNPs and 8 InDels were found between the two alleles from the purple parent pl102 and the green parent rt104 (Fig. 4).

Interestingly, such sequence variation was not found between the purple parent pll02 and another green cultivar gre101. The *BjMYB113* gene from green cultivar gre101 is almost identical to that from the purple cultivar, with only one SNP at -2,269 of promoter region (Fig. 4), and the *BjMYB113* gene from either cultivar does not have the 1,268-bp insertion in intron 1. BSR-seq analysis of the pl102 × gre101 segregating population also showed that the gene controlling leaf color was located in the same region as *BjMYB113* on chromosome J15 (Fig. S1) (see above). We then sequenced its flanking region and discovered an insertion of 1,033-bp fragment at 2,873 bp downstream its stop codon. Therefore, there are at least four alleles of the *BjMYB113* gene, including *BjMYB113a* (purple allele), *BjMYB113b* (green allele) and *BjMYB113c* (green allele with the 1,033-bp insertion in its 3’ downstream), as well as an allele identified previously with an insertion of 1,268-bp fragment in the first intron (Fig. 4).

### Overexpressing the *BjMYB113a, BjMYB113b, or BjMYB113c* alleles promoted anthocyanin accumulation in *Arabidopsis*

**Fig. 5:**
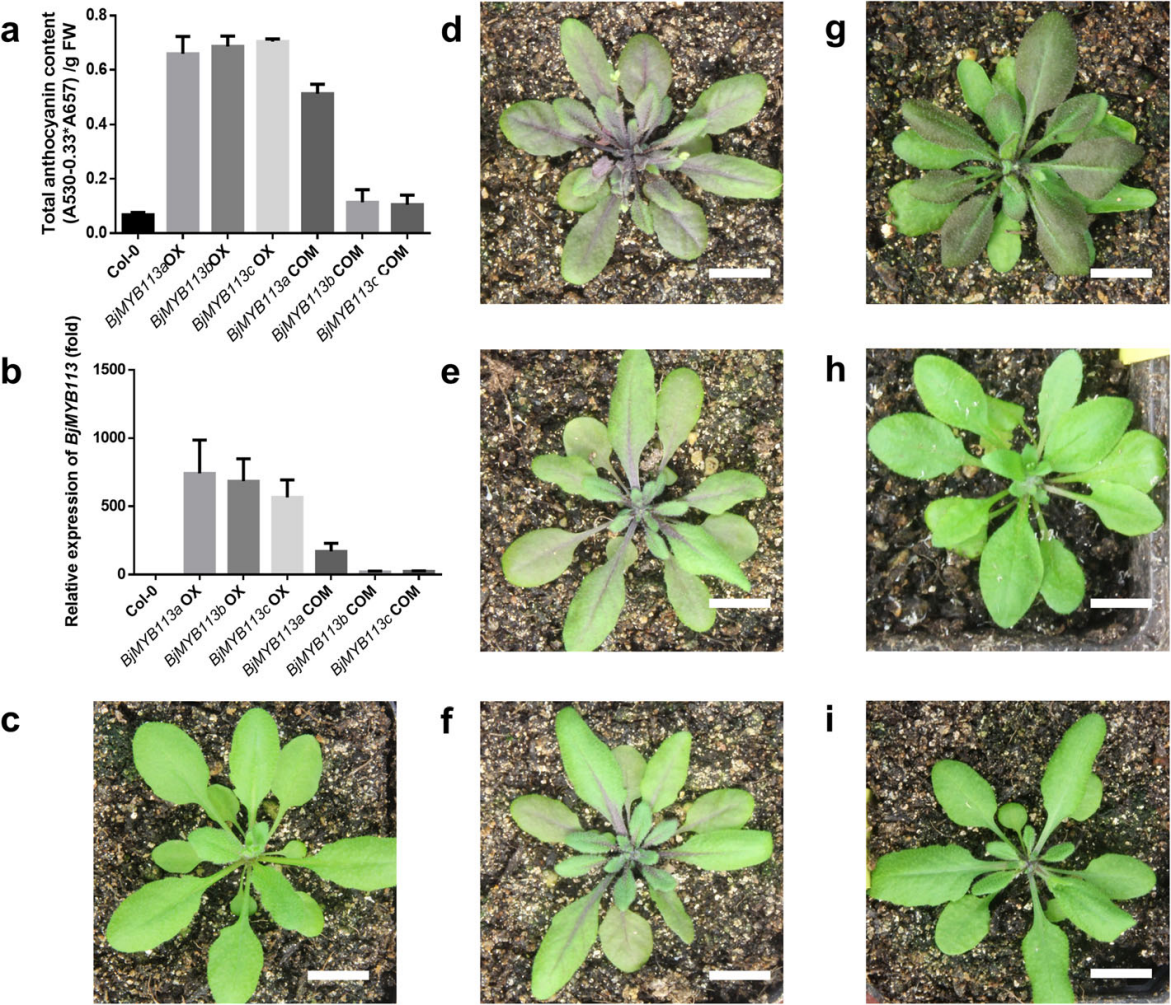
The total anthocyanin content and the expression of *BjMYB113* in *Arabidopsis* transgenic lines. **a** The total anthocyanin content in *Arabidopsis* transgenic lines. Col-0 is green wild-type *Arabidopsis* as the control, “*BjMYB113a* OX” refers to overexpression transgenic lines of *BjMYB113a* (CDS), “*BjMYB113a* COM” refers to complementary transgenic line of *BjMYB113a* (with native promotor and 3’region sequence). **b** The expression of *BjMYB113* in the *Arabidopsis* transgenic lines. **c** The wild-type *Arabidopsis* Col-0. **d-f** *BjMYB113a* (CDS), *BjMYB113b* (CDS) and *BjMYB113c* (CDS) overexpression transgenic lines. **g-i** *BjMYB113a*, *BjMYB113b* and *BjMYB113c* complementary transgenic line. Scale bar represents 1 cm

The coding sequence of the three alleles *BjMYB113a*, *BjMYB113b* and *BjMYB113c* are highly conserved in different mustard cultivars (Fig. S2). The coding sequence of *BjMYB113a* and *BjMYB113c* are identical, while *BjMYB113a* and *BjMYB113b* vary by three SNPs, with only one of them leading to amino acid change (M120K) (Fig. S2). To verify the function of these three *BjMYB113* alleles, their coding sequence were driven by cauliflower mosaic virus (CaMV) 35 S promoter and transferred into *A. thaliana* Col-0. All *Arabidopsis* lines overexpressing *BjMYB113* alleles had purple leaves and stems in comparison with green leaves and stems in wild type (Fig. 5c, d, e, f). As expected, the color change was due to high accumulation of anthocyanins and high expression of *BjMYB113* (Fig. 5a, b). Above results indicated that the proteins encoded by the three *BjMYB113* alleles were functionally identical. Therefore, the color variations of mustard cultivars were most likely caused by expression difference of the *BjMYB113* alleles.

### Promoter region but not coding region is responsible for the activation of the *BjMYB113a* gene

**Fig. 6:**
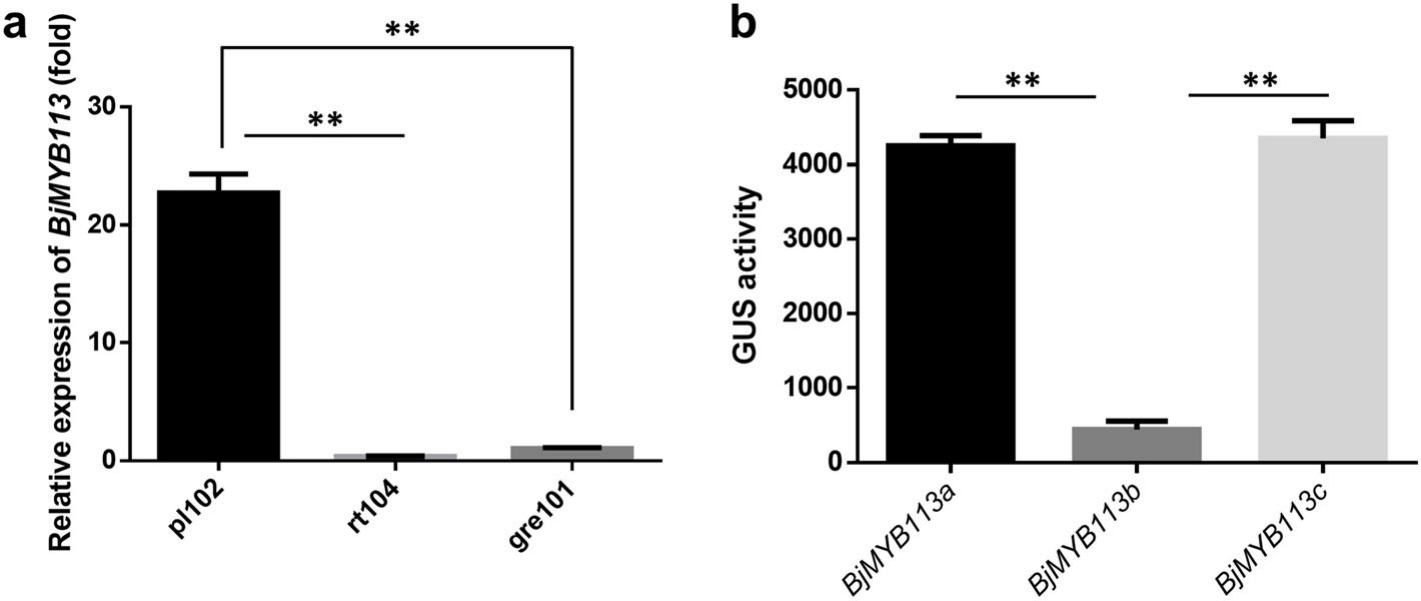
**a** The relative expression of *BjMYB113* in pl102, rt104 and gre101. **b** The promotor activity of *BjMYB113a*, *BjMYB113b* and *BjMYB113c.* Statistical analyzes were conducted by Student’s t test, **P < 0.01

We then investigated the expression level of the *BjMYB113* gene in different mustard cultivars. A quantitative real-time PCR analysis demonstrated that the expression level of *BjMYB113a* (from purple cultivar) was significantly higher than *BjMYB113b* and *BjMYB113c* (from green cultivars) (Fig. 6a). We hypothesize that the expression difference between alleles *BjMYB113a* and *BjMYB113b* was caused by their promoter regions, and the expression difference between alleles *BjMYB113a* and *BjMYB113*c was caused by the 1,033-bp insertion in downstream region of the latter.

To test above hypothesis, promoter activity of the three *BjMYB113* alleles was analyzed. *GUS* gene driven by the promoter of *BjMYB113a*, *BjMYB113b*, and *BjMYB113c*, respectively, was transformed into *Arabidopsis* Col-0. Six transgenic lines were obtained for each allele, and the GUS activities of these transgenic lines were measured. The GUS activity driven by promoter of *BjMYB113a* or *BjMYB113c* was significantly higher than that driven by the promoter of *BjMYB113b*, but no activity difference was observed between the promoters of *BjMYB113a* and *BjMYB113c* (Fig. 6b). Therefore, the polymorphisms in the promoter region accounts for the expression difference between *BjMYB113a* and *BjMYB113b*, and the low expression of *BjMYB113c* was likely caused by the 1,033-bp insertion in its downstream rather than the point mutation in its promoter region.

Forty-five SNPs and eight InDels were found between the promoter regions of *BjMYB113a* and *BjMYB113b*. The motif analysis was carried out for these two promoters. The activation of the *BjMYB113a* in purple cultivar pl102 was likely due to the point mutations at -1,317 (C to G), -1,576 (A to G), -1,588 (G to T), -2,593 (C to T). These four SNPs produced new transcription factor binding sites, including a MBSI motif (AAAAAAC(G/C)GTTA ), a bHLH motif (CANNTG), and two TATA-box motifs (ATTATA).

### The 1,033-bp insertion suppresses the expression of *BjMYB113c*

To verify the effects of the 1,033-bp insertion on the expression of *BjMYB113*, the *BjMYB113c* allele, including its native promoter and 3’region was transformed to *Arabidopsis* Col-0. No color change was observed in positive transgenic lines (Fig. 5i). In contrast, transgenic line of a construct containing *BjMYB113a* gene with its native promoter and 3’downstream sequences did change the color from green to purple (Fig. 5g). Note the main sequence difference between above two constructs was the 1,033-bp insertion. We conclude that high expression of *BjMYB113a* was due to mutations in its promoter region, and that the low expression of *BjMYB113c* was caused by the suppression effects of the 1,033-bp fragment inserted in its downstream region.

### High expression of the *BjMYB113a* activated the anthocyanin biosynthetic genes

**Fig. 7:**
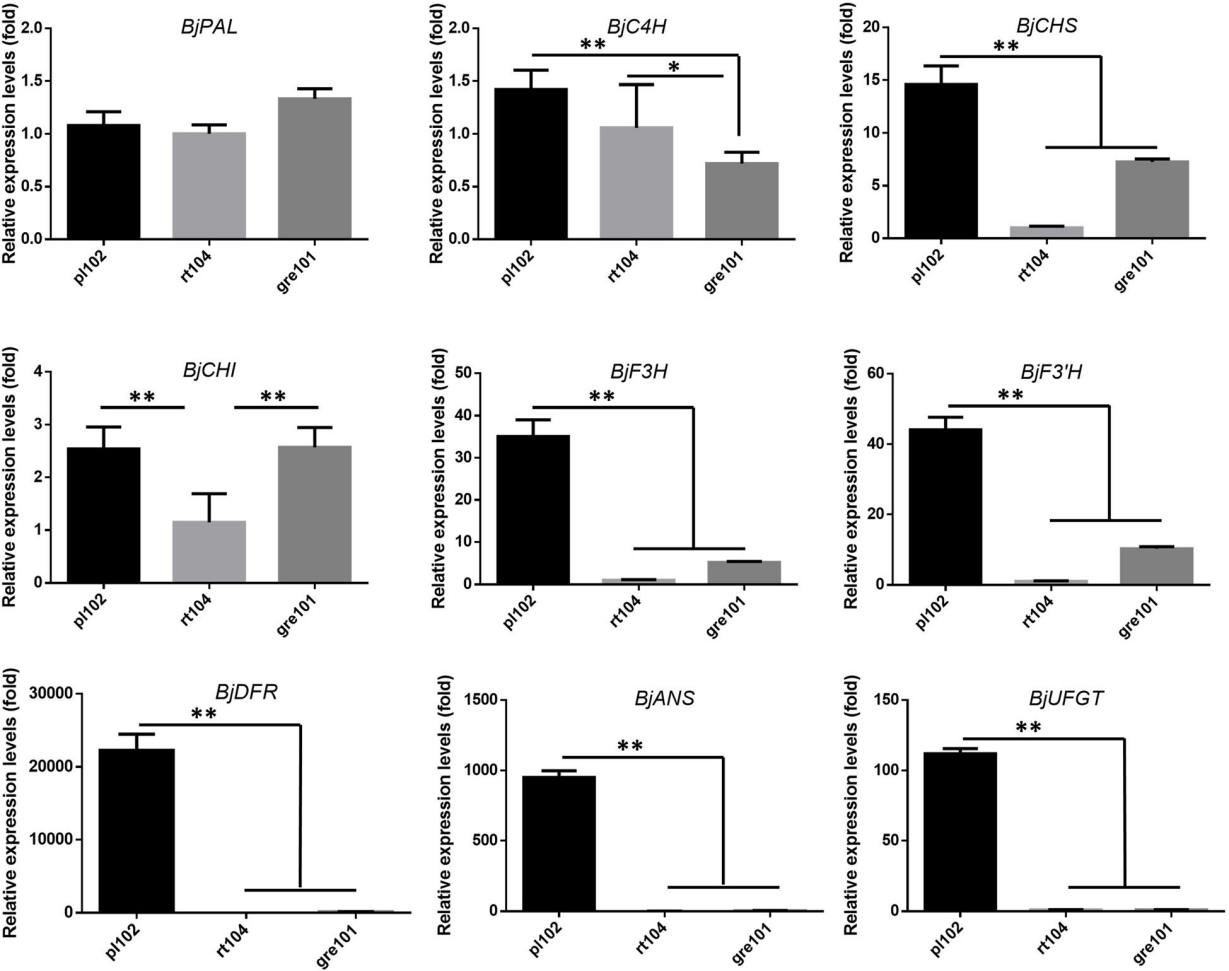
The relative expression of nine anthocyanin biosynthesis genes in three *Brassica juncea* cultivars pl102, rt104 and gre101. Statistical analyzes were conducted by Student’s t test, *P < 0.05; **P < 0.01

As an important component in the MBW transcription factor complex, activated *BjMYB113* gene may up-regulate multiple genes in the anthocyanin biosynthesis pathway. qRT-PCR was carried out to investigate the expression difference of genes, including *BjPAL* (*BjuA036480*), *BjC4H* (*BjuB015902*), *BjCHS* (*BjuA041225*), *BjCHI* (*BjuA004576*), *BjF3H* (*BjuA035478*), *BjF3’H* (*BjuA047311*), *BjDFR* (*BjuA033678*), *BjANS* (*BjuB044852*), and *BjUGFT* (*BjuA047199*). The qRT-PCR results showed that the expression of *BjCHS*, *BjF3H*, *BjF3’H*, *BjDFR*, *BjANS*, and *BjUGFT* in purple cultivar pl102 were significantly higher than that in green cultivars rt104 and gre101 (Fig. 7).

### The evolution of the *BjMYB113* gene

**Fig. 8:**
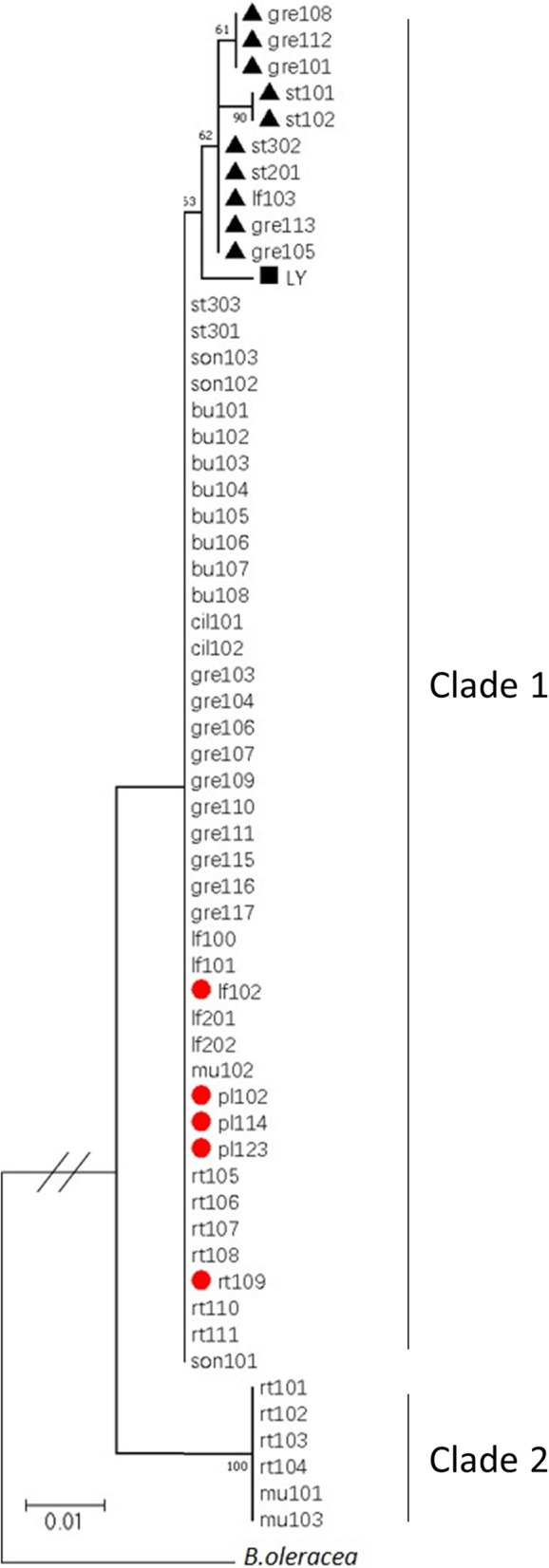
The neighbor-joining tree of the sequences of the *BjMYB113* alleles in 58 mustard accessions. The sequences of *BjMYB113* ortholog in *B. oleracea* was used as an outgroup. Red circle represents purple phenotypes, and others are all green phenotypes. Black triangle represents the 1,033-bp insertion in the *BjMYB113* gene in green mustards. Black rectangular represents the 1,268-bp insertion in the *BjMYB113* gene in green mustards

To understand the evolution of the *BjMYB113* gene in mustard, approximately 3 kb promoter sequences of 58 mustard accessions including 5 purple and 53 green ones were PCR amplified and sequenced. All accessions were also genotyped for the 1,268-bp insertion in intron 1 and the 1,033-bp insertion in its downstream. A neighbor-joining tree was constructed using the full-length gene sequences, with *BoMYB113* from *Brassica oleracea* as an outgroup (Fig. 8). Two major clades were found for these gene sequences from mustard, varying at least 50 nucleotides in promoter sequences between members from the two clades. The six sequences from Clade 2 are identical, all with the inactivated promoter as in green cultivar rt101 (*BjMYB113b*). The Clade 1 contains five distinct sequences including the sequence of *BjMYB113a*, which has the activated promoter and lacks the 1,033-bp insertion in its downstream. The second sequence (with three cultivars) is *BjMYB113c* with one nucleotide different from *BjMYB113a* in promoter sequence and has the 1,033-bp insertion in its downstream. The third sequence (two cultivars) varies only two nucleotides from *BjMYB113a* and also has the 1,033-bp insertion. The fourth sequence (five cultivars) has the same promoter sequence as *BjMYB113a* and with the 1,033-bp insertion. The fifth sequence (one cultivar LY) varies a 38-bp insertion in the promoter sequence and has the 1,268-bp insertion in intron 1. Therefore, all promoter sequences from Clade 1 are most likely activated ones (see above). Surprisingly, of the 52 cultivars in Clade 1, 36 have green leaves but they do not have the large insertions in either intron 1 or in its 3’ downstream. Three genotypes of 36 green cultivars were randomly chosen as representatives to investigate the expression level of *BjMYB113*. The expression level of *BjMYB113* from three green cultivars were significantly lower than *BjMYB113a* from purple cultivar, suggesting that there might be other mutation events leading to the inactivation of the *BjMYB113* gene or loss-of-function mutations in other genes of the anthocyanin biosynthesis pathway (Fig. S3).

## Discussion

### MYB plays a central role in color variation in plant species

In this study, we showed that the *BjMYB113* gene was responsible for the color variation in mustard, and point mutations and small InDels in the promoter region accounted for its activation and accumulation of the anthocyanins. Color polymorphisms might be caused by any genes in the anthocyanin biosynthesis pathways. However, the most frequent causal genes for natural color variations were MYB-encoding genes. For example, the *RLL2* gene which encodes an R2R3-MYB transcription factor, regulates multiple genes from the anthocyanin biosynthesis pathways and promote the high-level accumulation of anthocyanins in lettuce leaves [29]. In *B. oleracea* species, the *BoMYB2* gene has been activated independently for at least three times [31, 32]. The blood orange arose by insertion of a Copia-like retrotransposon to a MYB gene *Ruby* [26]. The *OsC1* mutations in the coding region vary degrees of apiculus coloration in rice [36].

While wild species in natural populations have green leaves, many horticultural cultivars have red/purple leaves, such as red leaf plum, purple lettuce and purple mustard. With the assumption that their wild ancestors had green leaves, the red or purple cultivars arose from mutations and artificially selected and maintained during domestication or breeding. We showed in this study that the *BjMYB113* gene was activated due to the accumulated point mutations and/or InDels. It will be interestingly to investigate which point mutation(s)/InDel(s) played the critical role in its activation.

In this study, we also found a mutation event that inactivated the *BjMYB113* gene, a large insertion in its downstream. BLASTN search of the inserted sequences showed that it had a high copy number in the genome of mustard. The inserted sequences do not show similarity to known transposable elements, and the sequences *per se* do not show a typical transposon structure such as target site duplication (TSD) or terminal inverted repeats (TIR).

The mechanism for the inactivation of the *BjMYB113* gene by the insertion remains unknown. One possibility is that the insertion may result in methylation in this region to downregulate its expression. We investigated cytosine methylation status in this region, but no significant difference in methylation ratio was found between *BjMYB113a* and *BjMYB113c* (Fig. S4). Another possibility is that the insertion disrupts an enhancer motif of the gene. It is also possible that the insertion might have altered the chromatin structure and consequently down-regulates gene expression in its vicinity.

Our sequence analysis suggested that at least 36 cultivars with green leaves have the activated promoter of the *BjMYB113a* allele from the purple cultivar, but they do not have the 1,033-bp insertion in its 3’ downstream or 1,268-bp insertion in intron 1 that could silence the gene. We sequenced the entire *BjMYB113* gene from some cultivars, but no sequences variations were identified. Therefore, these green phenotypes were most likely caused by loss-of-function mutations of other genes in the anthocyanin biosynthesis pathway rather than the *BjMYB113* gene.

In a recent report, a 1,268-bp insertion in the first intron suppressed the *BjMYB113* gene, leading to a low anthocyanin accumulation [3]. The only difference between the promoter sequences of the *BjMYB113* gene from that cultivar LY and the *BjMYB113a* allele is a 38-bp insertion, and it is still unclear whether the low expression of *BjMYB113* gene in the cultivar is due to the 38-bp insertion in its promoter or the insertion of the 1,268-bp in its intron 1 (Fig. 4).

### Different mutation events activating genes associated with anthocyanin biosynthesis

Many mutation events may activate genes associated with anthocyanin biosynthesis, including point mutations, insertions of transposable elements, gene duplications, and InDels in promoter region. Insertions of a CACTA-like transposon and a harbinger transposon activated *BoMYB2*, leading to anthocyanin accumulation in purple kale and purple cauliflower, respectively [31, 32]. A non-LTR retrotransposon activated *CaAn2* in purple *Capsicum annuum* [37]. A Copia-like retrotransposon activated *Ruby* leading to blood orange in *Citrus* [26]. Gene duplications and mutations in promoter region activated the *RLL2* gene leading to activated anthocyanin biosynthesis in lettuce [29]. The promoter variation of two *B* alleles determine distinct tissue specificities of anthocyanin production in maize [38].

### BSA in combination with RNA‑seq is an efficient method to map and clone causal genes

BSR-seq, an efficient and economic method, has been widely applied to identify genes that control important traits in many crops, including wheat [39], cabbage [40], watermelon [41], rice [42], cauliflower [43], and Chinese kale [44]. In this study, the *MPL* gene was also cloned through the BSR-seq analysis of F_2_ segregating populations. RNA-seq may not detect as many SNPs as DNA-sEq. However, in most cases, the number of SNPs identified by RNA-seq is enough for genetic analysis. Large difference of allele frequency of a SNP between two extreme pools provides direct evidence for the linkage between the SNP and a causal gene. The RNA-seq of the two pools also provides information on differentially expressed genes (DEGs). The DEGs, if located in the candidate region, might facilitate the identification of candidate gene. The GO analysis of the DEGs between the two extreme pools may also suggest the pathways that are associated with the trait of interest. In this study, the causal gene for the color variation in mustard, *BjMYB113*, showed expression difference between the two pools. Furthermore, several genes in the anthocyanin biosynthesis pathway were up-regulated in the purple pool compared with the green pool of the F_2_ segregating population. All these information greatly helped us pinpointing the *BjMYB113* gene as the causal gene and understanding the pathway involved for the color variation in mustard.

## Conclusions

We genetically cloned an R2R3-MYB coding gene controlling purple leaves in mustard. *BjMYB113* was activated due to promoter variations, leading to the high expression of genes in the anthocyanin biosynthesis pathway and the high accumulation of anthocyanin in the purple cultivar. However, a large insertion in its 3’region or in its first intron compromised the high expression in the active allele leading to green color. These results indicated that BjMYB113 transcription factor, as the important member of the MBW ternary complex, has experienced both gain-of-function and loss-of-function mutations during artificial selection and domestication. Our results contribute to a better understanding of the genetics and evolution of the *BjMYB113* gene controlling purple leaves and provide useful information for further breeding programs of mustard, which will be of broad interest to biologists.

## Methods

### Plant materials and genetic segregating populations

Purple mustard cultivar pl102 and green cultivars rt104 and gre101 were chosen to study the genetics of purple color leaves. These three mustard cultivars are brown mustard (*Brassica juncea*). Purple cultivar pl102 was crossed with green cultivars rt104 and gre101, respectively, to develop two F_2_ segregating populations. These F_2_ segregating populations along with its parents were acquired from professor Hanhui Kuang’s lab (College of Horticulture and Forestry Sciences, Huazhong Agricultural University) in this study. Parental seed lines were originally acquired at National Center for Vegetable Improvement (Central China). Seeds were germinated and seedings grew in a greenhouse under 16/8 h photoperiod at 25 °C ± 2 °C. All plants were planted to the field on campus of Huazhong Agriculture University, Wuhan, China.

### Analysis of total anthocyanin contents

Method of total anthocyanin content analysis followed the guidelines and protocols described previously [45]. For anthocyanin extraction, 0.1 g tissues of each sample were incubated in 300 µL of extraction buffer (methanol containing 1 % HCl) overnight at 4 °C in the dark. After extraction, 200 µL of water and 200 µL of chloroform were added to each sample, and absorbances were read at 530 and 657 nm. The quantity of anthocyanin was determined by calculating absorbance at 530 nm (A530) – 0.33A657, and each sample was extracted and measured in three independent experiments.

### Flavonoid metabolite profiling

The relative quantities of flavonoid metabolites in *Brassica juncea* leaf samples were analyzed with a liquid chromatography-electrospray ionization-tandem mass spectrometry (LC-ESI-MS/MS) system by MetWare (Wuhan, China). The liquid chromatography–electrospray ionization-tandem mass spectrometry (LC-ESI-MS/MS) system was used for the relative quantification of anthocyanin metabolites in *Brassica juncea* leaves samples. The protocols were described detailedly in Methods S1.

### Bulked Segregant Analysis and RNA‑seq (BSR‑seq)

Bulked Segregant Analysis in combination with RNA-seq (BSR-seq) was used to map genes controlling purple leaves [46]. A total of 20 purple individuals from an F_2_ segregating population were mixed as the purple-pool, and 20 green individuals were mixed as the green-pool. Total RNAs were extracted from the two pools using RNAiso plus (Takara, Japan). RNA-seq was performed on Illumina Hiseq2500 platform (Novogene, China), and approximately 5 GB clean data were obtained for each pool. The data were mapped to *Brassica juncea* reference genome [47], using the Bowtie software [48]. SNP callings were performed using SAMtools [49]. Low-quality SNPs with map quality value < 30, reads depth < 10× or base quality value < 20 were excluded. The key parameter of Δ(SNP-index) was used to identify the target region for purple/green traits [50]. The Δ(SNP-index) was calculated by subtracting the SNP-index value of the green-pool from the SNP-index value of purple-pool. Cleaved amplified polymorphic sequence (CAPS) markers were designed in the candidate region, and were used to screen the population to fine map the casual gene. The primers used in map-based cloning were shown in Table S3. The RNA-seq data supporting the results of this study is available in the NCBI SRA (Sequence Read Archive, http://www.ncbi.nlm.nih.gov/sra/) repository under BioProject PRJNA672814.

### RNA extraction and quantitative Real-Time reverse transcription polymerase chain reaction (qRT-PCR) analysis

Total RNAs were extracted from leaves using RNAiso plus (Takara, Japan). The cDNA was synthesized using TransScript One-Step gDNA Removal and cDNA Synthesis SuperMix (TransScript, China) with Oligo-dT18 primer. qRT-PCR analysis followed the guidelines and protocols described previously [51]. All reactions were performed using the SYBR Premix (5.0 µL of 2× SYBR Premix Go Taq II, 0.5 µL of primers, 1.0 µL of cDNA, and 3.5 µL of ddH2O). Melting curve analysis of qRT-PCR samples revealed that there was only one product for each gene primer reaction. The PCR products were sequenced to confirm the specific amplification. A house-keeping gene *BjEF-1-α*^*37*^ was used as an internal standard in tissues. The relative expression levels were counted using the formula 2^−△△Cq^ as described in Bio-Rad protocol, and statistical differences were calculated using student’s test. Three biological replications and three technical replications were performed in qRT-PCR. The primers used in qRT-PCR analysis were shown in Table S3.

### Plasmid construction and plant transformation

The full-length *BjMYB113* cDNA was cloned into the pRI101-GFP vector with CaMV 35 S promoter to construct the overexpression vector. The full-length *BjMYB113* gene sequence (including native promoter and 3’region sequence) was amplified and cloned into the pRI101-GFP to construct the complementary vector. Approximately 3 kb promoter region of three *BjMYB113* alleles were amplified and recombined into the pCAMBIA1301-GUS vector for checking GUS activity. All vectors were constructed using homologous recombination. Positive plasmids were verified by sequencing and then transformed into *Agrobacterium tumefaciens* GV3101 using thermal stimulation method. The vectors were transformed into *Arabidopsis* using floral-dip method [52]. All primers used for vector construction were shown in Table S3.

### Promoter activity analysis

The quantitative GUS activity was measured using the Lu’s methods with slight modification [53]. GUS activity was detected in 1-month-old *Arabidopsis* leaf tissues (10 mg) from three independent transgenic lines and six individuals in each line. Total proteins were extracted using 300 µL GUS extraction buffer (50 mM phosphate buffer, pH 7.0; 10 mM EDTA, pH 8.0; 0.1 % Sodium Dodecvl Sulfate; 10 mM β-mercaptoethanol). BCA Protein Assay (Beyotime Biotechnology, China) was used to measure the protein concentrations. Extraction (100 µL) was added to 900 µL GUS extraction buffer containing 1 mM 4-methylumbelliferyl glucuronide (MUG, Sigma) and incubated at 37 °C. The 900 µL stop solution (1 M Sodium Carbonate) immediately added into 100 µL the above reaction mixture and 60 min later, respectively. Fluorescence of 4-methylumbelliferone (MU) was monitored using Tecan Infnite™ at 455 nm emission and 365 nm excitation. GUS activity was expressed as µmoles 4-methylumbelliferone (MU) min^− 1^ mg^− 1^ protein.

### Sequence analysis and neighbor-joining tree

The promoter sequence of *BjMYB113* alleles was analyzed using PlantCARE (http://bioinformatics.psb.ugent.be/webtools/plantcare/html/).

Sequence alignments were conducted using Muscle program and manually adjusted in GeneDoc (http://www.nrbsc.org/gfx/genedoc/). The neighbor-joining tree was constructed using MEGA 7.0 [54] and bootstrap values were calculated using 1,000 times.

The gene sequences (*BjMYB113a*, -*b*, and -*c*) supporting the results of this study are available in the NCBI GenBank (https://www.ncbi.nlm.nih.gov/genbank/) under accession number MW166171- MW166173 of BankIt2394807. The primers used in gene sequences analysis were shown in Table S3.

### Methylation sensitive digestion

Method of methylation sensitive digestion followed the guidelines and protocols described previously [55]. Genomic DNA was extracted from purple mustard cultivar pl102 and green cultivar gre101. The quality and integrity of extracted genomic DNA was evaluated by spectrophotometric analysis using NanoDrop (ThermoFisher Scientific, U.S.A.). Genomic DNA was digested by the methylation-sensitive endonuclease McrBC according to the manufacturer’s instructions (New England Biolab Inc., U.S.A.). Then, qRT-PCR analysis was performed [51]. Each sample was measured in three independent experiments. The mean C_T_ values were used to calculate ΔC_T_ as follows: ΔC_T_ = [C_T_(McrBC treatment) – C_T_(Control)] and the methylation percentage was calculated as methylation% = 100 – (100 × 2^−ΔC^_T_).

## Supplementary Information

**Additional file 1: Figure S1. **Map-based cloning of the *MPL *gene in F_2_ population of pl102 × gre101.

**Additional file 2: Figure S2. **The alignment of protein sequences of *BjMYB113 *and its most similar R2R3-MYB transcription factors homologous genes.

**Additional file 3: Figure S3. **The relative expression of *BjMYB113* of purple cultivar pl102 and three green cultivars.

**Additional file 4: Figure S4. **Analysis of the methylation status in the 3’ regions of* BjMYB113a* and *BjMYB113c*.

**Additional file 5: Table S1. **Different types of anthocyanin and their concentrations in purple and green cultivars with three biological replications.

**Additional file 6: Table S2. **The Chi-square (χ2) of two purple/green segregating populations.

**Additional file 7: Table S3. **The primers used in this research.

**Additional file 8: Method S1. **Anthocyanin metabolite profiling.

## Data Availability

The data supporting the result of this study are within the paper and its additional files. All sequencing datasets are deposited in the National Centre for Biotechnology Information (NCBI) under the BioProject ID PRJNA672814 with the Sequence Read Achieve (SRA) accession SRR12919405-SRR12919408. The gene sequences (*BjMYB113a*, -*b*, and -*c*) supporting the results of this study are available in the NCBI GenBank (https://www.ncbi.nlm.nih.gov/genbank/) under accession number MW166171 - MW166173 of BankIt2394807.

## Abbreviations

ANS: Anthocyanidin synthase
BSR-seq: Bulked Segregant Analysis and RNA-seq
CaMV: Cauliflower mosaic virus
CAPS: Cleaved amplified polymorphic sequence
CHI: Chalcone isomerase
CHS: Chalcone synthase
DEGs: Differentially expressed genes
DFR: Dihydroflavonol 4-reductase
F3H: Flavanone 3-hydroxylase
GO: Gene Ontology
InDels: Insertions and Deletions
LC-ESI-MS: Liquid Chromatography-Electrospray Ionization-Mass Spectrometry
MPL: Mustard Purple Leaves
MU: 4-methylumbelliferone
MUG: 4-methylumbelliferyl glucuronide
ORFs: Open reading frames
PAL: Phenylalanine-ammonia lyase
qRT-PCR: Quantitative Real-Time reverse transcription polymerase chain reaction
ROS: Reactive oxygen species
SNPs: Single nucleotide polymorphisms
TIR: Terminal inverted repeats
TSD: Target site duplication
