# Supplementary figures and images for: Frequent gain- and loss-of-function mutations of the BjMYB113 gene accounted for leaf color variation in Brassica juncea
Source: BMC Plant Biol. 2021 Jun 29;21:301. doi: 10.1186/s12870-021-03084-5 (PMC8240407; doi:10.1186/s12870-021-03084-5)

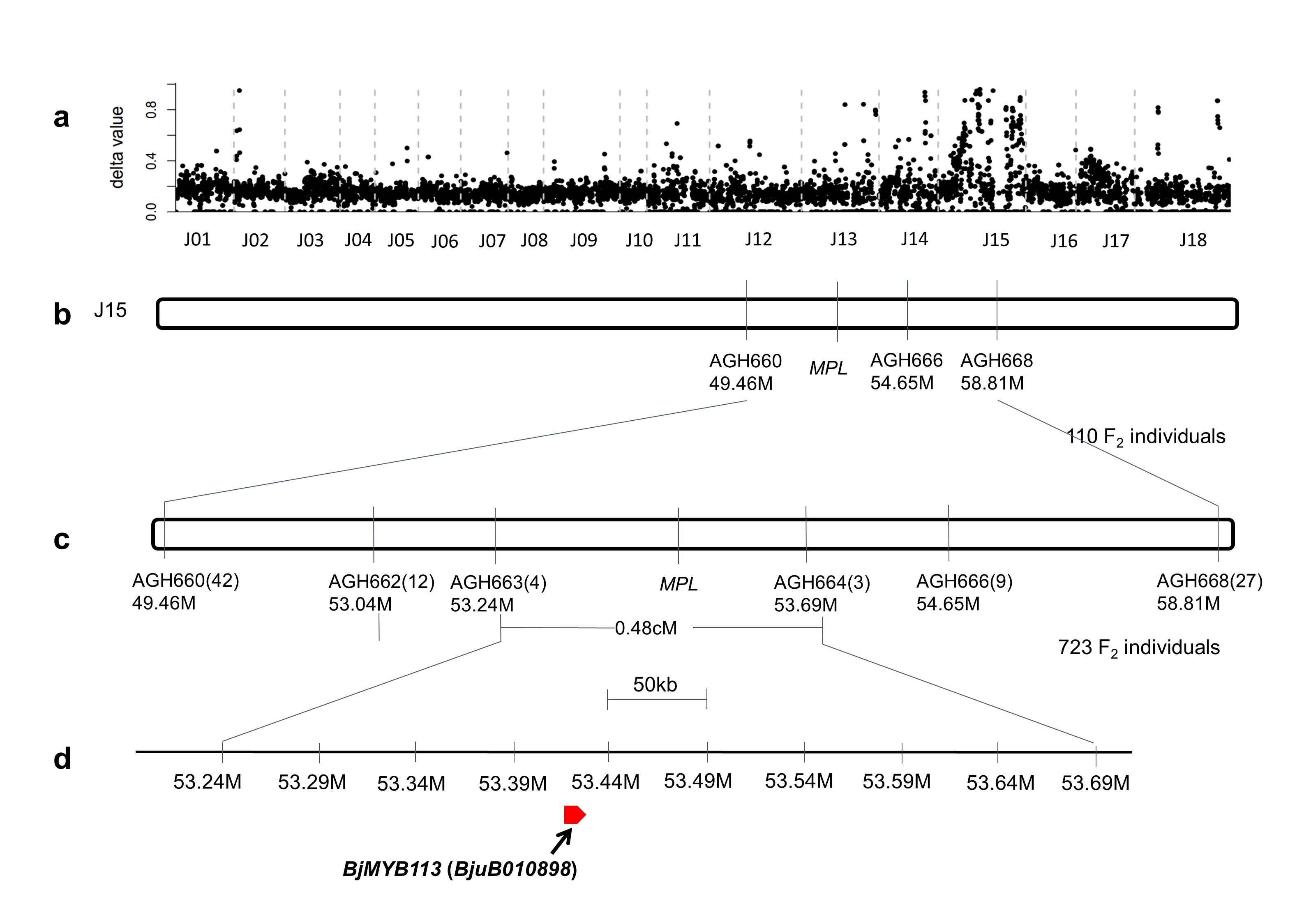

Supplement: Supplementary file 1 — Additional file 1: Figure S1. Map-based cloning of the MPL gene in F2 population of pl102 × gre101. [file 12870_2021_3084_MOESM1_ESM.jpg]

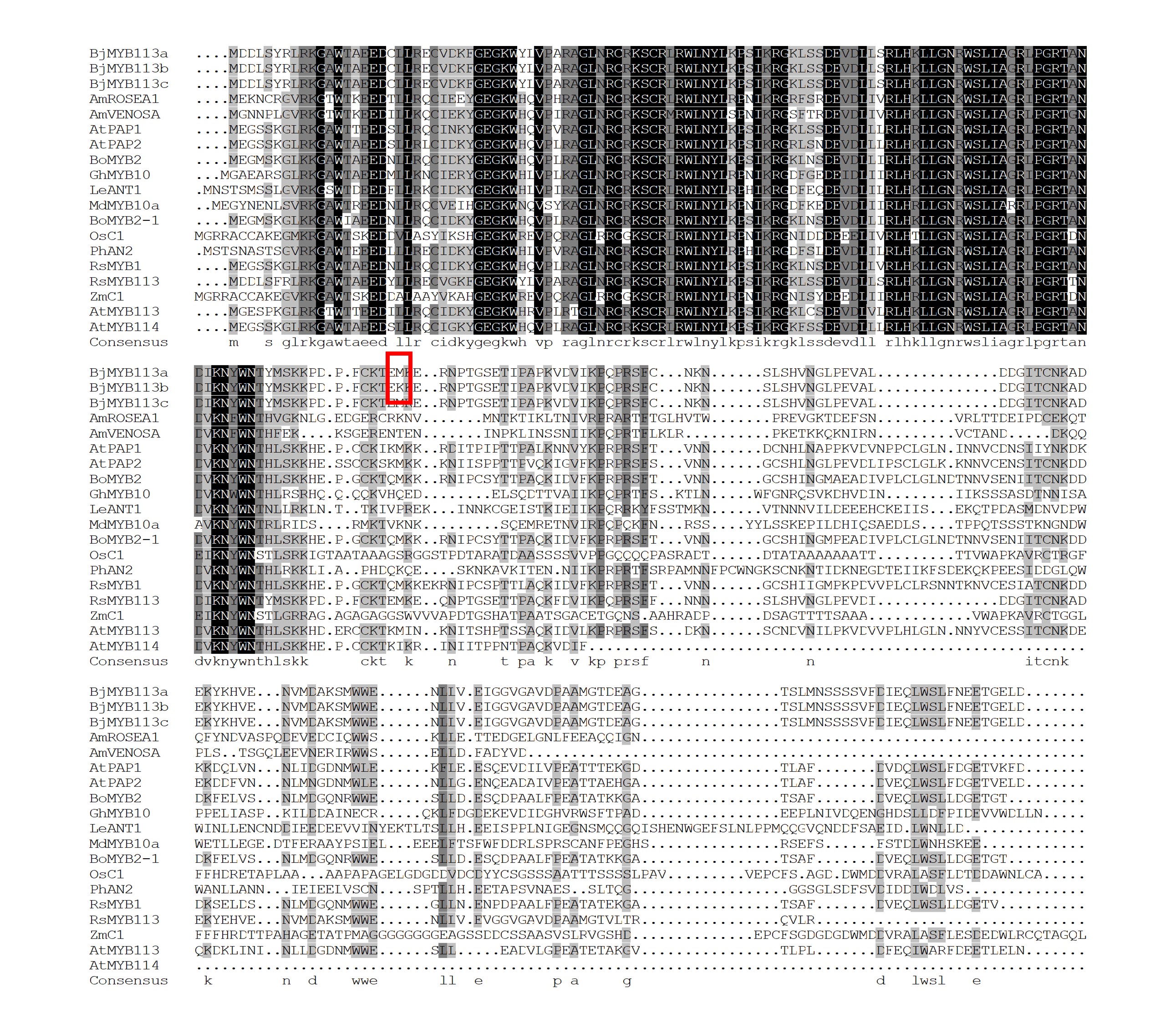

Supplement: Supplementary file 2 — Additional file 2: Figure S2. The alignment of protein sequences of BjMYB113 and its most similar R2R3-MYB transcription factors homologous genes. [file 12870_2021_3084_MOESM2_ESM.jpg]

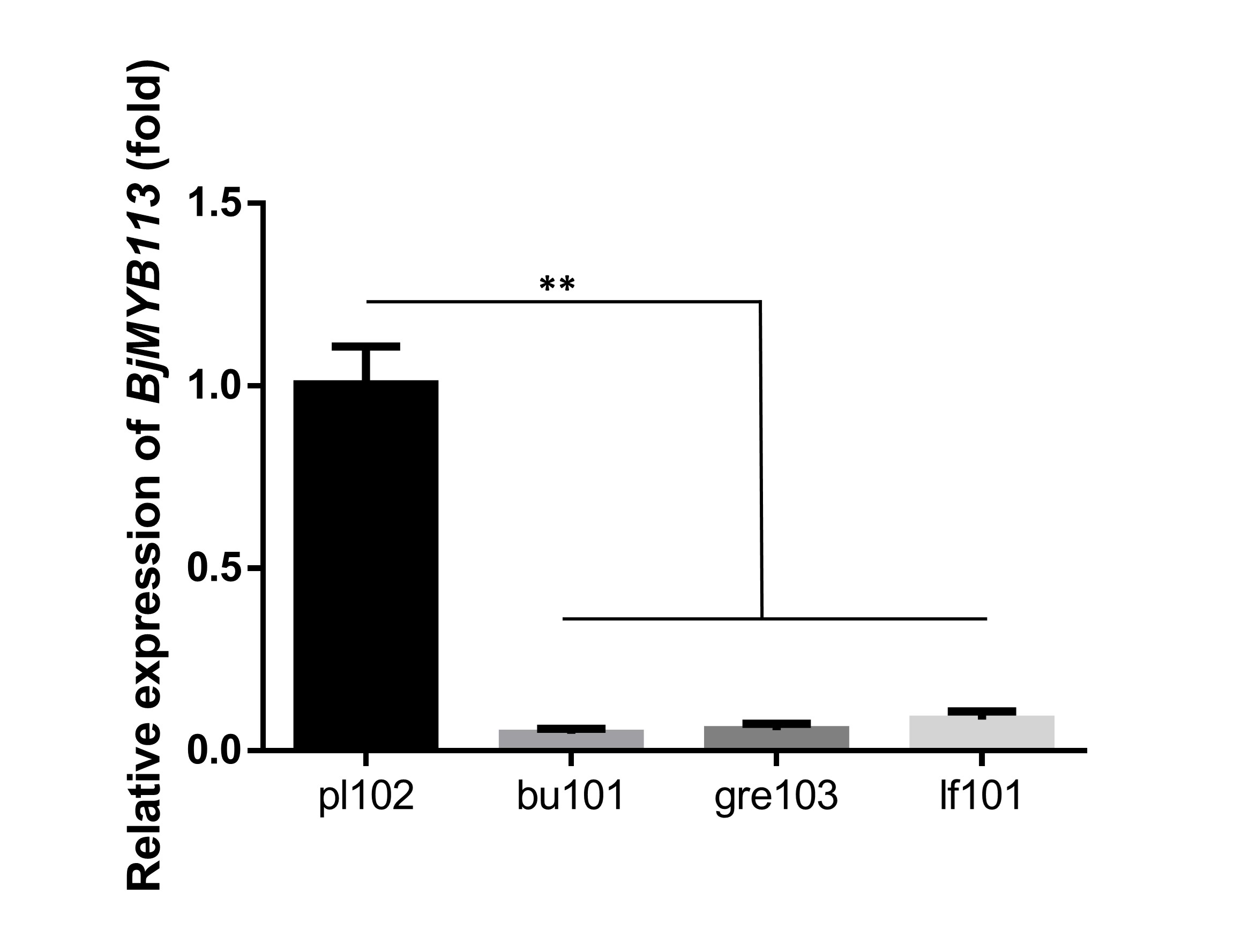

Supplement: Supplementary file 3 — Additional file 3: Figure S3. The relative expression of BjMYB113 of purple cultivar pl102 and three green cultivars. [file 12870_2021_3084_MOESM3_ESM.jpg]

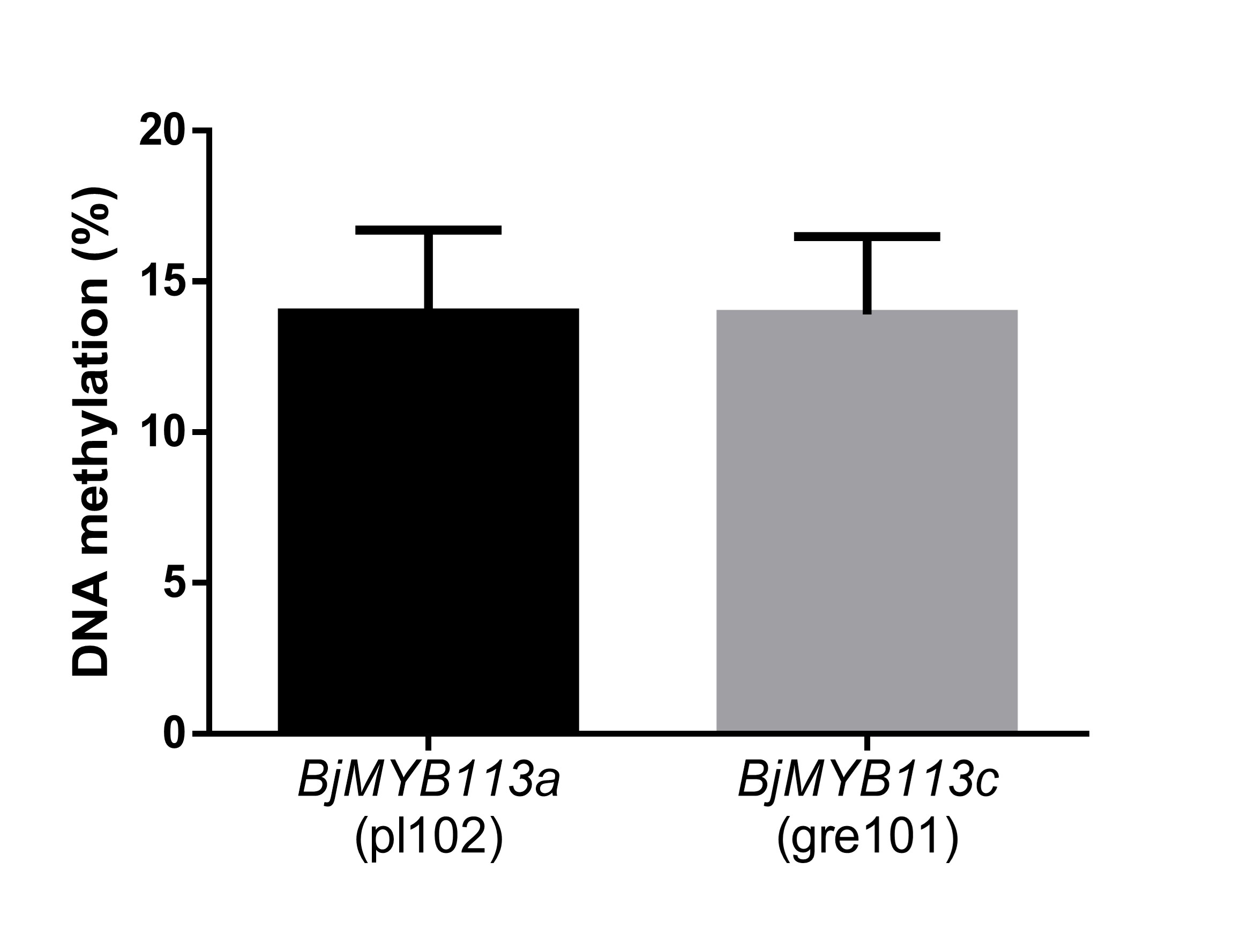

Supplement: Supplementary file 4 — Additional file 4: Figure S4. Analysis of the methylation status in the 3’ regions of BjMYB113a and BjMYB113c. [file 12870_2021_3084_MOESM4_ESM.jpg]
