# Supplementary material for: Frequent gain- and loss-of-function mutations of the BjMYB113 gene accounted for leaf color variation in Brassica juncea
Source: BMC Plant Biol. 2021 Jun 29;21:301. doi: 10.1186/s12870-021-03084-5 (PMC8240407; doi:10.1186/s12870-021-03084-5)
Supplement: Supplementary file 8 — Additional file 8: Method S1. Anthocyanin metabolite profiling. [file 12870_2021_3084_MOESM8_ESM.docx]

**Method S1**

**Anthocyanin metabolite profiling**

The relative quantities of flavonoid metabolites in *Brassica* *juncea* leaves samples were analyzed with a liquid chromatography-electrospray ionization-tandem mass spectrometry (LC-ESI-MS/MS) system by MetWare (Wuhan, China). The liquid chromatography–electrospray ionization-tandem mass spectrometry (LC-ESI-MS/MS) system was used for the relative quantification of anthocyanin metabolites in *Brassica* *juncea* leaves samples. The *Brassica* *juncea* leaves samples (0.1 g powder) were treated with 1.0 mL 70% aqueous methanol overnight at 4 ℃. After a centrifugation at 10,000 × g for 10 min, the supernatants were collected (CNWBOND Carbon-GCB SPE Cartridge, 250 mg, 3 mL; ANPEL, Shanghai, China), filtered (SCAA-104, 0.22 μm pores; ANPEL), and analyzed with an LC-ESI-MS/MS system comprising the Shim-pack UFLC CBM30A system (Shimadzu) and the 6500 Q TRAP system (Applied Biosystems). The analytical conditions were as follows: LC: column, Waters ACQUITY UPLC HSS T3 C18 (1.8 µm, 2.1 mm × 100 mm); solvent system, water (0.04% acetic acid):acetonitrile (0.04% acetic acid); gradient program, 100:0 (v/v) at 0 min, 5:95 (v/v) at 11.0 min, 5:95 (v/v) at 12.0 min, 95:5 (v/v) at 12.1 min, and 95:5 (v/v) at 15.0 min; flow rate, 0.40 mL/min; temperature, 40 °C; injection volume: 2 μL. The effluent was analyzed with an ESI-triple quadrupole-linear ion trap (Q TRAP) system. The LIT and triple quadrupole (QQQ) scans were acquired on a 6500 Q TRAP LC-MS/MS system equipped with an ESI Turbo Ion-Spray interface. The system was operated in the positive ion mode and controlled with the Analyst 1.6.3 program (AB Sciex). Instrument tuning and mass calibration were completed with 10 and 100 μmol/L polypropylene glycol solutions in QQQ and LIT modes, respectively. The QQQ scans were acquired as MRM experiments with the collision gas (nitrogen) set to 5 psi. The DP and CE were optimized for individual MRM transitions. A specific set of MRM transitions was monitored for each period according to the metabolites eluted within the period. Differentially accumulated metabolites were identified in each sample pair [fold-change ≥ 2 or ≤ 0.5, variable importance ≥ 1].
